# Supplementary material for: Severe Muscle Deconditioning Triggers Early Extracellular Matrix Remodeling and Resident Stem Cell Differentiation into Adipocytes in Healthy Men
Source: Int J Mol Sci. 2022 May 14;23(10):5489. doi: 10.3390/ijms23105489 (PMC9143135; doi:10.3390/ijms23105489)
Supplement: Supplementary file 1 [file ijms-23-05489-s001.zip › ijms-1711465-supplementary.pdf]

We declare the same experimental design was already published in: "Early deconditionning of human skeletal muscle and the effects of a thigh cuff countermeasure" by Fovet et al. (doi.org/10.3390/ijms222112064). We declare reusing our previous published data that permit to validate the Dry Immersion model, i.e, muscle fiber CSA and laminin staining, whole leg MRI data, and knee extension isometric torque.

## Materials and Methods:

### Magnetic resonance imaging (MRI)

Siemens-Avanto device was used in CHU Rangueil in Toulouse, France to performed images. Subjects were transferred to MRI room keeping -6° head-down bed rest position to simulated microgravity in the best way. Imaging was recorded 4 days bedore immersion and directly after. Briefly, the mark was located exactly halfway between the left anterior superior iliac spine and the upper part of the ipsilateral (left) patella.

The measurements were taken on the left side because of the biopsy on the right side. This condition was chosen to avoid inducing heterogenic signals in MRI analysis of the biopsy site. The surface area was calculated by OsiriX MD software (v.7.0.1. 64-bit software). The surface area of the anterior compartment of the thigh was measured in the same region of interest (ROI) on images centred on the same landmark. The measurements were repeated until agreement was within the 3% threshold, and the values were averaged.

### Maximal voluntary isometric torque measurement

Knee extension isometric torque was assessed using Con-Trex device (Physiomed; Schnaittach, Germany). Measurement were performed on left lower limb, 4 days before the start of DI protocol and directly after the 5 days of DI during the upright phase. Before starting protocol, participants were familiarized with the equipment and protocol. Maximal isometric torque was determined at 80° extension of the knee. Briefly, participants were firmly attached in seated position to avoid parasite movements. Protocols was composed of short warm-up in neutral position then series of extension – isometric maintain contraction and flexion movement were performed. Each isometric contraction was maintained 5-7 seconds and two minutes of recovery were allowed after 3 sets of measurement. Maximal strength was recorded on every sets to determined maximal isometric torque. The total duration of maximal voluntary isometric torque measurement was approximately 15 minutes

### Muscle cross-sectional area quantification

First, to evaluate VL atrophy, the muscle fiber cross-sectional area (CSA) was measured in transverse cryosections (10 µm thick) of the Pre-DI and Post-DI VL biopsies. Serial transverse cryosections, cut using a cryostat at -25°C, were dried and fixed in acetone for 10 minutes before washes in phosphate-buffered saline (PBS), blocking, and permeabilization with 0.1% Triton-X100 and 20% horse serum. Sections were incubated with an anti-MyHC antibody (Table 1) at 37°C for 1 hour, and then with the relevant secondary antibody (Table 1) at 37°C for 1 hour. This was followed by incubation with an anti-laminin antibody (Table 1) at 37°C for 1 hour, rinses in PBS, and incubation with the relevant secondary antibody (Table 1) at 37°C for 1 hour.

Nuclei were stained with Hoechst solution (1/1000) for 30 seconds. Then, sections were mounted with Permafluor® (Cat# : TA-030-FM, Epredia, Thermo fisher scientific, USA) and dried at RT overnight. Images were obtained using a ZEISS Axio Scan (INM, Montpellier) at x20 magnification with a focus on the DAPI-stained nuclei. The fiber size (calculated on an average of 440 fibers) and signal quantification were analysed using ImageJ (version 1.46).

## Results:

### Dry immersion induces muscle atrophy and strength loss

DI is a fast and drastic deconditioning model, leading to a significant reduction in muscle mass and strength [44]. The present DI protocol was designed to gain insight into the first few days of muscle disuse during which muscle atrophy develops rapidly [43]. Indeed, at the end of the 5 days DI period (Figure S1), the knee maximal isometric voluntary contraction (MVC) for extension was decreased by -14% in the Control (215 Nm Pre-DI vs. 185 Nm Post-DI, $p<0.05$) and by -10% in the Cuffs group (220 Nm Pre-DI vs. 198 Nm Post-DI, $p<0.05$), (Figure S2a) (Fovet et al. [31]). The mean VL fiber CSA also was decreased by -16.5% in the Control (3372 µm² ± 952 Pre-DI vs. 2814 µm² ± 368 post-DI, $p<0.05$) and by -20.5% in the Cuffs group (3593 µm² ± 1005 Pre-DI vs. 2858 µm² ± 952 post-DI, $p<0.05$) (Figure S2b-c), any differences between fibers type were observed [31]. Moreover, the whole leg muscle CSA, measured by MRI was also reduced (-2.5% vs Pre-DI; $p<0.05$) (Figure S2d). These results highlight the fast atrophy induced by DI.

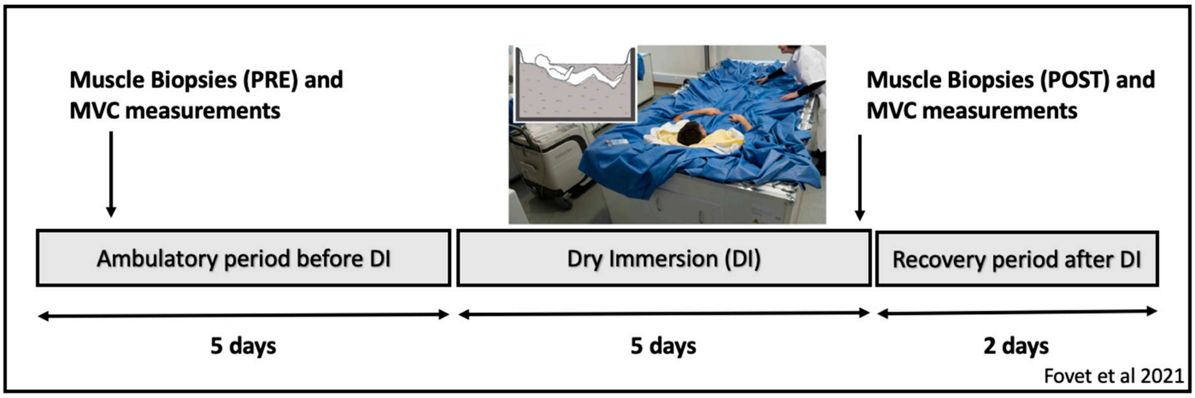


**Fig S1** Experimental protocol. *Participants arrived in the evening 5 days before dry immersion (DI) initiation and left 2 days after DI in the morning. The experimental protocol included four days of ambulatory baseline measurements before DI, five days of DI, and two days of ambulatory recovery. Baseline (pre-DI) and post-DI measurements included muscle biopsy of the vastus lateralis, and maximal voluntary contraction (MVC) measurement. Participants randomized in the Cuffs group wore thigh cuffs during the 5 days of DI, from 10h to 18h the first day and from 8h to 18h the other days, at a counter-pressure of 30-50 mmHg. Reprinted with permission Fovet et al 2021[31]*

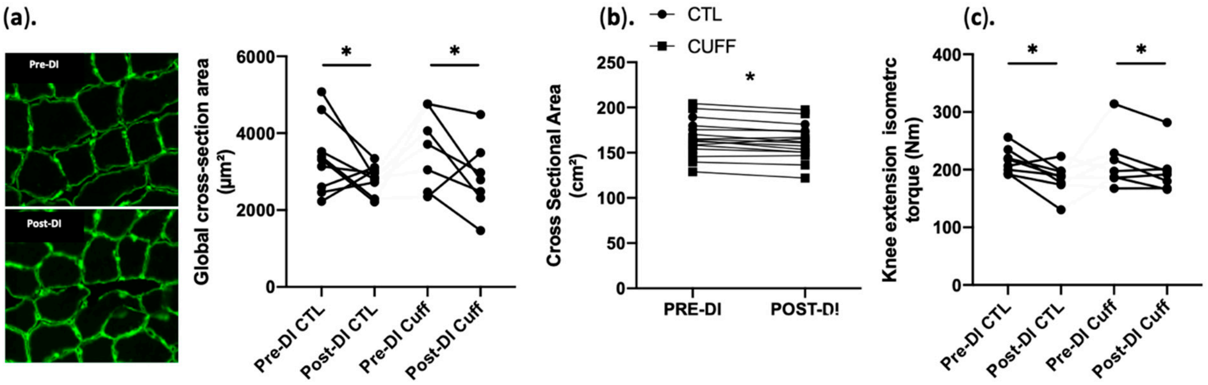


**Fig S2** *(a) Myofiber cross-sectional area measurement and staining (laminin) in Pre-DI and Post-DI vastus lateralis biopsies. (b) Whole leg (anterior loge + posterior loge) cross sectional area (cm²) measured by MRI before (Pre-DI) and after (Post-DI) 5 days of dry immersion (c) Maximal voluntary knee extension isometric torque (Nm) before (Pre-DI) and after (Post-DI) 5 days of dry immersion. CTL, participants who underwent DI without thigh cuffs (n=9); Cuffs, participants who wore thigh cuffs during DI (n=9). *p<0.05. Two way ANOVA with repeated measure was performed*
